# Strength and Conditioning Practices of Brazilian Olympic Sprint and Jump Coaches

**DOI:** 10.5114/jhk/159646

**Published:** 2023-01-20

**Authors:** Irineu Loturco, Thomas Haugen, Tomás T. Freitas, Chris Bishop, Tulio B. M. A. Moura, Valter P. Mercer, Pedro E. Alcaraz, Lucas A. Pereira, Anthony Weldon

**Affiliations:** 1 NAR—Nucleus of High Performance in Sport, São Paulo, Brazil.; 2 Department of Human Movement Sciences, Federal University of São Paulo, São Paulo, Brazil.; 3 University of South Wales, Pontypridd, Wales, United Kingdom.; 4 School of Health Sciences, Kristiania University College, Oslo, Norway.; 5 UCAM Research Center for High Performance Sport, Catholic University of Murcia (UCAM), Murcia, Spain.; 6 Faculty of Sport Sciences, Catholic University of Murcia (UCAM), Murcia, Spain.; 7 Faculty of Science and Technology, London Sports Institute, Middlesex University, London, UK.; 8 Centre for Life and Sport Sciences, Birmingham City University, Birmingham, United Kingdom.

**Keywords:** athletic performance, track and field, coaching practices, speed, resistance training, athletes

## Abstract

Olympic coaches are likely to have adequate knowledge and implement effective training programs. This study aimed to describe and critically examine the strength and conditioning practices adopted by Brazilian Olympic sprint and jump coaches. Nineteen Olympic coaches (age: 50.2 ± 10.8 years; professional experience: 25.9 ± 13.1 years) completed a survey consisting of eight sections: 1) background information; 2) strength-power development; 3) speed training; 4) plyometrics; 5) flexibility training; 6) physical testing; 7) technology use; and 8) programming. It was noticed that coaches prioritized the development of explosiveness, power, and sprinting speed in their training programs, given the specific requirements of sprint and jump events. Nevertheless, unexpectedly, we observed: (1) large variations in the number of repetitions performed per set during resistance training in the off-season period, (2) a higher volume of resistance training prescribed during the competitive period (compared to other sports), and (3) infrequent use of traditional periodization models. These findings are probably related to the complex characteristics of modern competitive sports (e.g., congested competitive schedule) and the individual needs of sprinters and jumpers. Identification of training practices commonly used by leading track and field coaches may help practitioners and sport scientists create more effective research projects and training programs.

## Introduction

Track and field athletes are usually distinguished by their exceptional physical performance in different events, which regularly occur under maximum effort conditions (e.g., sprinting and jumping) (Franceschi et al., 2020; Haugen et al., 2019b). These “superior capabilities” seem to be even more pronounced during the Olympic Games, when coaches and practitioners use all resources available to optimize the competitive potential of their athletes (Olusoga et al., 2012; Pohl et al., 2019). Each discipline has its own features and rules, with some being more dependent on endurance-related factors (e.g., 5,000- and 10,000-m) and others on strength-, speed-, and power-related variables (e.g., 100-m dash and long jump) (Ben-Zaken et al., 2015; Loturco et al., 2015b). Although all Olympic athletes are at the limit of human performance, the margin for error during explosive events is usually much smaller as athletes have a very short time (i.e., ~10 s in the 100-m dash) or a reduced number of trials (i.e., three attempts in long jumps) to properly execute the techniques and achieve their best performances (Loturco et al., 2018b; Tonnessen et al., 2013).

Accordingly, variations in performance obtained by top-level sprinters and jumpers across their professional careers tend to be minimal. For example, decreases inferior or equal to 8% have been observed in elite sprinters from the age of 18 to their personal-best times (Freitas et al., 2021; Haugen et al., 2015). In addition, annual improvements in the range of only 0.1–0.2% have been detected for both sprinters and jumpers during their early 20s (Haugen et al., 2018). Despite these modest (but meaningful) changes in performance, the body of evidence related to training, testing, technique, and biomechanics of sprinting is vast and robust, with a large number of recent and “classic” studies examining these factors (Bezodis et al., 2019; Colyer et al., 2018; Haugen and Buchheit, 2016; Haugen et al., 2019b; Healy et al., 2019; Loturco et al., 2021b; Mcmaster et al., 2014; Morin et al., 2011; Walker et al., 2021). Consequently, track and field coaches have numerous resources at their disposal for enhancing coaching skills and strategies and hence, optimizing the competitive level of their sprinters (or track and field athletes who heavily rely on sprinting speed, such as long jumpers) (Bridgett and Linthorne, 2006; Granić and Pavlović, 2021; Ngetich and Rintaugu, 2013). Under this rationale, it is highly expected that Olympic sprint and jump coaches are more likely to implement evidence-based practices in their daily routines. Nevertheless, few studies to date have analyzed or reported the coaching practices of Olympic coaches, especially with a focus on their strength and conditioning (S&C) methods.

This knowledge is even more scarce in developing countries (e.g., Brazil), where access to technology, expensive equipment, and good training facilities is usually limited, which may compromise the implementation of more sophisticated training approaches (Loturco et al., 2022). Analysis of training and testing practices regularly employed by these highly-specialized practitioners could provide valuable and useful insights to coaches of lower-level athletes (i.e., regional or national) and younger age categories, as well as allow them to reflect on the effectiveness of their own training processes (Haugen, 2021). Therefore, the purpose of this study was to investigate, describe, and critically examine the S&C practices commonly adopted by Brazilian Olympic sprint and jump coaches.

## Methods

### Participants

Nineteen Brazilian Olympic sprint and jump coaches (age: 50.2 ± 10.8 years, age range: 32– 73 years; professional experience: 25.9 ± 13.1 years, range: 10–50 years), participated in this study. Coaches on average participated in 4 ± 3 (range: 1– 9) Olympic Games, working with 7 ± 7 (range: 1– 23) athletes. Athletes training under their supervision won ten Olympic Games, 12 World Championship, 7 Diamond League, and 42 Pan-American Games medals. Regarding the academic background of coaches, 5% held a Ph.D. degree, 21% had a master’s degree, 53% had completed a post-graduate course, and all of them were graduated in Physical Education or Sport Science. The study was conducted under the ethical standards of the Helsinki Declaration and was approved by the local Ethics Committee.

### Study Design

This cross-sectional descriptive study was designed to characterize the common training and testing practices of Brazilian sprint and jump coaches. Given that these coaches are generally encouraged to implement contemporary research and science-informed practices, it is important to establish if this is the case. For this purpose, a survey used in previous studies to assess the practices of coaches from distinct countries and sport disciplines (Loturco et al., 2022; Weldon et al., 2021a; Weldon et al., 2020; Weldon et al., 2021b) was adapted and designed using Typeform^TM^. The survey consisted of eight sections (1—background information; 2—muscular strength and power development; 3—speed development; 4— plyometrics; 5—flexibility; 6—physical testing; 7— technology use; and 8—programming) comprising 27 fixed responses and 15 open-ended questions. In one specific question regarding the use or not of periodization strategies in their training programs, coaches were allowed to provide additional comments or justify their responses at will. As certain questions allowed more than one response, some questions included more responses than others. During the survey preparation, four experienced track and field coaches completed the survey and minor adjustments were made to the wording and structure of some questions, to ensure they were clear and appropriate for the surveyed population. A complete explanation of the general information necessary to complete the survey, the study purpose, and the confidentiality of information and identity were provided on the first page. Thereafter, participants provided consent and anonymously completed the online survey.

### Data Acquisition and Analyses

The survey responses were downloaded from Typeform^TM^ into a customized spreadsheet. Fixed response questions were assessed using frequency analysis and open-ended questions using a thematic analysis approach (Braun and Clarke, 2006), via the following processes: 1) familiarization with the data, 2) generating initial codes, 3) searching for themes, 4) reviewing themes, 5) defining and naming themes, and 6) producing the report. This thematic-analysis method has been used in previous studies surveying coaches (Loturco et al., 2022; Weldon et al., 2021a; Weldon et al., 2021b). Subsequently, key themes representing the main ideas emerging from the raw data were generated for open-ended questions. Some open-ended responses provided information for multiple topics, which could be grouped and considered for further analysis. All topics were reviewed and agreed upon by all authors.

## Results

### Muscular Strength and Power Development

**Figure 1:**
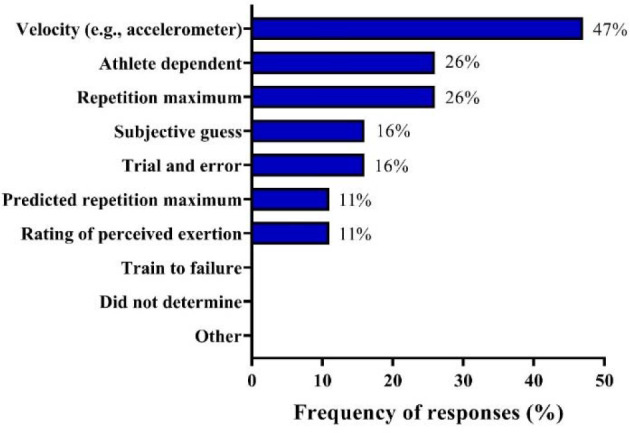
Load determination procedures used during strength-power training sessions by Brazilian Olympic sprint and jump coaches.

Table 1 shows the frequency of responses regarding the organization of strength training programs during preparatory and competitive periods. Figure 1 depicts responses to how coaches determined set loads during strength training sessions. Table 2 demonstrates absolute and relative results regarding the use or not of periodization strategies in strength training programs. Table 3 shows the average recovery time prescribed between strength/power training sessions, sport-specific training, and competitions. Additionally, coaches were asked whether they used Olympic weightlifting and associated derivatives in their programs. Responses demonstrated that 79% of coaches used the snatch, 58% the clean, 47% the clean & jerk, 16% the power clean, and 5% the power snatch, while 5% did not use these techniques. The most commonly reported resistance training methods were variable (79%), eccentric (63%), concentric (58%), machine (53%), isometric (37%), and isoinertial (16%). Table 4 provides the ranking of the five most important exercises that coaches used in their strength training programs.

**Table 1:** Absolute and relative (%) frequency of responses regarding the organization of the strength training program during preparatory (PP) and competitive (CP) periods (n = 19).

		1	2	3	4	5	>5	Other
Weekly sessions	PP	0 (0)	4(21)	9(47)	4(21)	0(0)	2(11)	0(0)
	CP	4(21)	9(47)	3(16)	2(11)	0(0)	0(0)	1(5)
		0–15	16–30	31–45	46–60	61–75	>75	Other
Session’s length (min)	PP	0(0)	0(0)	2(11)	7(37)	8(42)	2(11)	0(0)
	CP	0(0)	2(11)	6(32)	7(37)	2(11)	1(5)	1(5)
		1–2	3–4	5–6	7–8	9–10	>10	Other
Sets per exercise	PP	1(5)	13(68)	3(16)	0(0)	0(0)	1(5)	1(5)
	CP	1(5)	15(79)	0(0)	0(0)	0(0)	0(0)	3(16)
Repetitions per exercise set		1–3	4–6	7–9	10–12	13–15	>15	Other
	PP	0(0)	6(32)	3(16)	6(32)	1(5)	2(11)	1(5)
	CP	4(21)	11(58)	3(16)	0(0)	0(0)	0(0)	1(5)

**Table 2:** Responses regarding models of the strength training plan over the season (n = 19).

	Absolute (n)	Relative (%)
Through the use of periodization models that follow preplanned and/or fixed routines, selecting some events as “most important events/competitions” where athletes must achieve peak performance.	6	31.6
Through the use of programs constantly readjusted according to the individual or collective physical and physiological responses, not necessarily following fixed routines, trying to maintain high performance levels during all/multiple events/competitions of the season.	10	52.6
Other	3	15.8

**Table 3:** Absolute and relative (%) frequency of responses regarding the average recovery time between distinct training sessions (n = 19).

	Same day	24 h	36 h	48 h	>48 h
Recovery time between strength-power training and sport-specific training	6(32)	6(32)	1(5)	3(16)	3(16)
Recovery time between strength-power training and competition	1(5)	4(21)	3(16)	3(16)	8(42)
Recovery time between sport-specific training and competition	1(5)	1(5)	1(5)	3(16)	13(68)

**Table 4:** Ranking of the five most important exercises used in strength training programs (n = 19).

Order of importance	Exercises	n (%)
1	Squat and variations	8(42)
	Olympic weightlifting and derivatives	5(26)
	Ballistics Hip thrust	3(16) 1(5)
	Lunge	1(5)
	Did not specify	1(5)
2	Squat and variations Olympic weightlifting and derivatives Ballistics Did not specify	11(58) 4(21) 2(11) 2(11)
3	Olympic weightlifting and derivatives Ballistics Bench press Calf raises Hip thrust Leg curl Squat and variations Stiff-leg deadlift Did not specify	8(42) 2(11) 1(5) 1(5) 1(5) 1(5) 1(5) 1(5) 3(16)
4	Ballistics	4(21)
	Olympic weightlifting and derivatives	3(16)
	Squat and variations	3(16)
	Hip thrust	1(5)
	Lunge	1(5)
	Core exercises	1(5)
	Did not specify	6(32)
5	Calf raises Hip thrust Lunge Squat and variations Core exercises Leg extension Olympic weightlifting and derivatives Did not specify	2(11) 2(11) 2(11) 2(11) 1(5) 1(5) 1(5) 8(42)

### Speed Development

**Figure 2:**
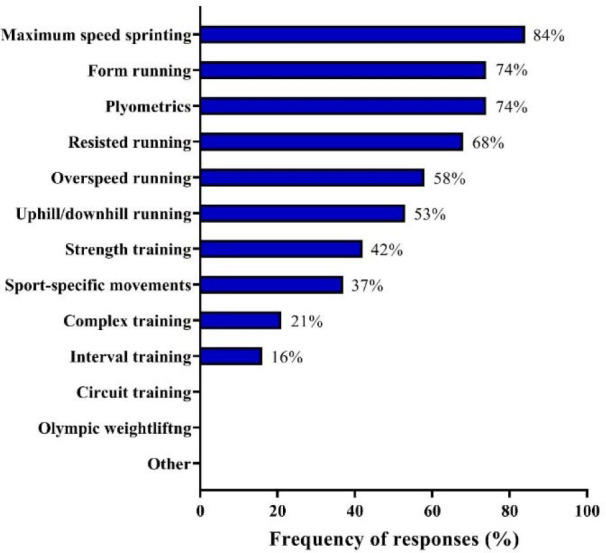
Methods for speed development used by Brazilian Olympic sprint and jump coaches.

Figure 2 depicts responses regarding the methods most used by coaches for speed development. The methods most frequently reported were maximum speed sprinting (84%), form running (i.e., technical drills) and plyometrics (both 74%), resisted running (68%), and overspeed running (58%).

### Plyometrics

**Figure 3:**
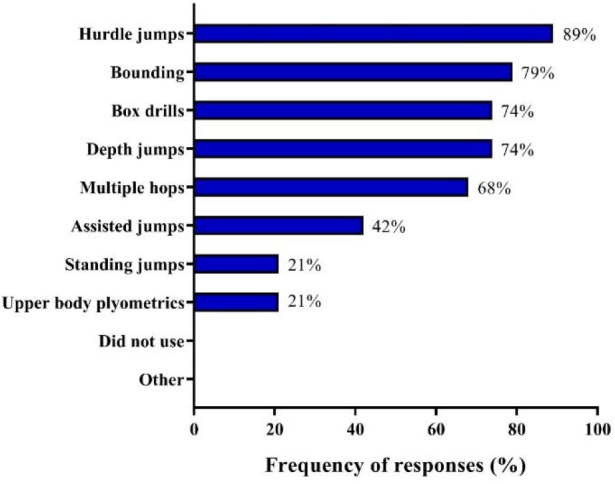
Plyometric exercises used by Brazilian Olympic sprint and jump coaches.

Coaches were asked the main reasons why they implemented plyometric exercises in their programs, with speed development (89%) being the most reported, followed by improving jump ability (68%), lower-body power (63%), injury prevention (26%), total-body power (16%), and upper-body power (11%). Regarding the period of the season that coaches usually employed plyometric training, preparatory (68%) and competitive (63%) periods were most frequently reported, followed by all year round (37%). Regarding the integration of plyometrics into their training schedule, 53% of coaches reported that it was used on separate days and after resistance training, 42% of coaches applied plyometric exercises as part of complex training, and 26% before resistance training. Figure 3 depicts the frequency of responses involving plyometric exercises commonly used by coaches in their programs.

### Flexibility Development

Sprint and jump coaches were asked to report when athletes were encouraged or required to perform flexibility exercises in their program. After training (37%) was the most frequent response, followed by before training (32%), and during training (11%). The most common forms of flexibility training used by coaches were dynamic (84%), active and proprioceptive neuromuscular facilitation (both 53%), ballistic (47%), static (42%), passive (21%), and isometric (11%). The average duration of a typical flexibility session was 11–15 min (37%), >21 min (26%), 16–20 min (21%), and 6– 10 min (5%), whereas 11% reported “other”.

### Physical Testing

**Figure 4:**
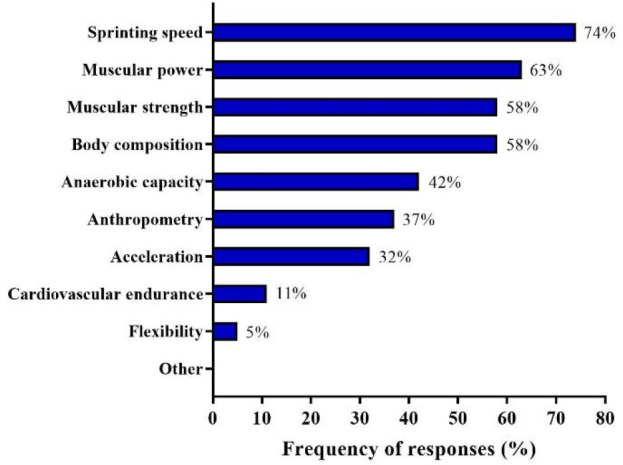
Physical tests employed by Brazilian Olympic sprint and jump coaches.

The most common time reported by coaches for physical testing of athletes was all year round (68%), followed by (only during) the preparatory period (21%). The competitive period, the off-season, and no testing were selected by 5% of coaches. Figure 4 shows the frequency of responses regarding the physical tests commonly used by sprint and jump coaches. Furthermore, coaches were asked how they monitored athletes’ well-being, with verbal questionnaires (79%) being most reported, followed by written questionnaires (16%) and online questionnaires or mobile applications (both 11%), whereas 5% did not monitor their athletes’ well-being.

### Technology Use

**Figure 5:**
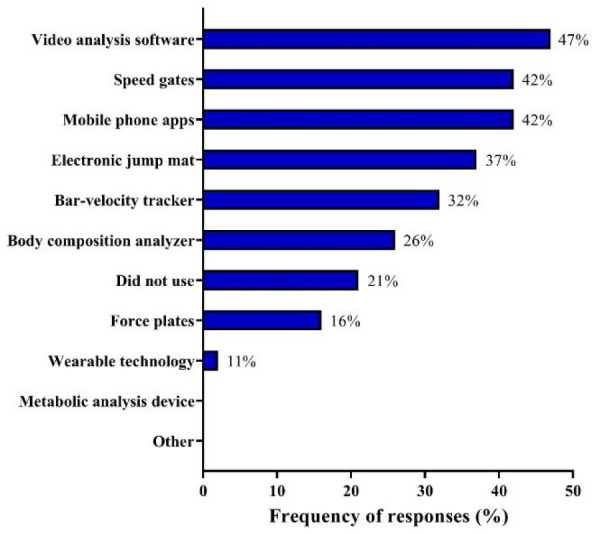
Technology-based equipment utilized by Brazilian Olympic sprint and jump coaches.

Figure 5 depicts responses regarding the technology-based equipment that coaches used in their training programs. Video analysis software (47%) was the most frequent response, followed by speed gates and mobile phone apps (both 42%). Other frequent responses included the use of electronic jump mats (37%) and bar-velocity trackers (32%).

### Programming

Coaches were asked to report the most significant issues faced during their training practices. Responses included the lack of equipment and poor training facilities (47%), financial problems and athletes’ commitment (both 16%), long commutes (11%), and the competitive calendar (5%), whereas 5% did not report any such issues. Another question was to report whether coaches felt there was anything unique about their training program, with most coaches reporting “no” (63%), while 37% answered “yes”. Some affirmative responses were: *“alternative places to perform training sessions in specific periods of the season”, “communication with the athlete”, “good agreement between theory and practice”, “movement perfection based on technical refinement”*, and *“persistency”*. In addition, coaches were asked whether they employed strategies to individualize training loads according to the characteristics of each athlete, with 95% answering “yes” and 5% “no”. When asked whether they would change something in their training program, given unlimited time and resources, 89% answered “yes”, while 11% answered “no”. For coaches who selected “yes”, some responses involved *“higher participation in international training camps and competitions”*, and *“better use of technology and equipment for training load control and physical assessment”*. Finally, the last question was about their opinion on future trends in sprint and jump training practices, with technological advancement (26%) being the most reported, followed by better training monitoring approaches (21%), better interaction with multidisciplinary professionals (16%), better understanding of metabolic markers, higher training intensity, and evolution of the science (all 5%), while 21% did not present any opinion.

## Discussion

In this study, we examined the S&C training and testing practices of Brazilian Olympic sprint and jump coaches. As expected, according to the specific technical and physical requirements of these track and field disciplines, elite sprint and jump coaches typically prioritized the development of explosiveness, power, and sprinting speed in their training programs (Haugen et al., 2019b; Healy et al., 2021; Koyama et al., 2011). Overall, the results of this survey support these assumptions, but, at the same time, reveal some novel and interesting findings which are probably related to the demanding, challenging, and complex characteristics of modern competitive sports (Kiely, 2018; Loturco and Nakamura, 2016; Schwellnus et al., 2016; Soligard et al., 2016). Below we will present and discuss each of these topics and their possible implications in a point-by-point manner.

### Resistance Training Prescription

#### Preparatory Period (Off-Season Phase)

A substantial number of sprint and jump coaches (47%) prescribed three resistance training sessions per week. Approximately 21% of them utilized two or four weekly sessions, whereas only 11% implemented >5 sessions per week. For 42% and 37% of coaches, typical resistance training sessions lasted between 61 and 75 min or between 46 and 60 min, respectively. The majority of them (68%) used 3–4 sets per exercise, with only a minority using 5–6 sets (16%), and >10 sets or 1–2 sets (5% for both set configurations). Thirty-two % of coaches prescribed 10–12 or 4–6 repetitions per set; 16% 7–9 repetitions; 11% more than 15 repetitions; and 5% of them implemented sets with 13–15 repetitions. According to the present findings, a “typical” resistance training session for Brazilian Olympic sprint and jump coaches during the preparatory period occurs between 2 and 4 times per week, lasts more than 60 min, and comprises 3–4 sets of 4–6 or 10–12 repetitions per set. This large variation in the number of repetitions per set is seemingly a result of the training conception: while some coaches are more concerned with structural and morphological changes (which require a higher number of repetitions per set), others are more interested in inducing neural adaptations, prescribing sets within the “common strength-power zones” (i.e., 4–6 repetitions) (Cormie et al., 2011b; Hartmann et al., 2015).

In general, the volume of resistance training commonly employed by these track and field coaches across the preparatory period was higher than that observed in recent surveys of other coaching populations (e.g., soccer, cricket, and rugby S&C coaches) (Weldon et al., 2022), who, although prioritizing a similar training frequency (2–4 sessions per week), predominantly prescribed resistance training sessions lasting up to 45 min (Loturco et al., 2022; Weldon et al., 2021b) or between 45 and 60 min (Jones et al., 2016; Weldon et al., 2021a). In a study conducted with National Basketball Association (NBA) S&C coaches, Simenz et al. (2005) also found a high (but not superior) percentage of coaches who frequently applied resistance training sessions lasting more than 60 min during the off-season. However, this rate was similar to that observed for sessions lasting 46–60 min (40% of NBA S&C coaches, for both 46–60 and >60-minute sessions). Hence, the predominance of extended resistance training sessions (>60 min) across preparatory periods appears to be exclusive to this group of “explosive” track and field athletes. To some extent, this can be attributed to the key role played by strength and power qualities in sprint and jump events (Haugen et al., 2019b; Healy et al., 2021; Koyama et al., 2011) and, consequently, the necessity of maximizing these “essential” capabilities as a basis for more specific and intensive training interventions (which are usually programmed for the subsequent training phases) (Bompa and Buzzichelli, 2019). In this regard, Brazilian Olympic sprint and jump coaches seem to dedicate a considerable amount of time of their off-season training programs to develop and optimize these neuromuscular abilities in their athletes. Somewhat surprisingly, this tendency was not limited to this training period, but was also observed during the in-season phase.

#### Competitive Period (In-Season Phase)

Almost half of coaches (47%) prescribed two resistance training sessions per week within the competitive period; 21%, 16%, and 11% of them applied 1, 3, or 4 weekly training sessions, respectively. The most reported duration for resistance training sessions was 46–60 min (37%), 31–45 min (32%), 16–30 and 61–75 min (11%), and >75 min (5%). The majority of coaches (79%) implemented training sessions with 3–4 sets, with only a minority using 1–2 sets (5%). Finally, the most prescribed set ranges throughout this phase comprised 4–6 (58%), 1–3 (21%), and 7–9 (16%) repetitions per set. Overall, a “typical” resistance training session for Brazilian Olympic sprint and jump coaches across the in-season was performed twice a week, lasted from 46 to 60 min, and involved 3–4 sets of 4–6 repetitions per exercise.

As previously mentioned, our findings suggest that these coaches also prescribed greater volumes of resistance training during the competitive period (when compared to S&C coaches from other sports, such as cricket and soccer) (Loturco et al., 2022; Weldon et al., 2021a; Weldon et al., 2021b). This highlights the importance of developing and maintaining high levels of strength and power in these explosive and highly specialized track and field athletes. Nonetheless, this volume of strength-power training may be similar or even inferior to that observed in surveys conducted with coaches of combat (i.e., wrestling) (Jamshidi et al., 2014) and “contact” disciplines (i.e., ice-hockey and rugby) (Ebben et al., 2004; Jones et al., 2016), which could be justified by the specific needs of these sports. Both combat and contact sports require not only substantial strength-power levels, but also a considerable amount of muscle mass to cope with high-impact forces experienced in combats (e.g., powerful strikes and takedowns) (Marinho et al., 2016) and matches (e.g., tackles in rugby) (Gabbett et al., 2011). This certainly demands a great deal of time and effort from coaches and athletes during the in-season phase, thus resulting in longer and more frequent resistance training sessions. On the other hand, interestingly, the number of repetitions per set commonly prescribed within this training period seems to be similar among these sports, with both Brazilian Olympic sprint and jump coaches and, for example, wrestling and ice-hockey coaches prioritizing strength-power sets up to 4–6 repetitions (usually performed with heavy loads or at higher velocities) (Ebben et al., 2004; Jamshidi et al., 2014).

Therefore, specifically with regard to the track and field coaches surveyed in this study, there was a clear trend towards increased intensity (and decreased volume) throughout the in-season phase. This tendency can be attributed to the considerable reduction in total training time as a consequence of the programmed (e.g., tapering) and mandatory (e.g., journeys and official competitions) activities that commonly occur over this congested period (Schwellnus et al., 2016; Soligard et al., 2016). As such, sprint and jump coaches implement this resistance training structure (i.e., lower volume and higher intensity) not only to maintain and improve the strength and power levels achieved in the off-season (a methodological approach used by S&C coaches from different sports) (Weldon et al., 2022), but also to increase neuromuscular readiness and diminish perceptual fatigue across successive competitions. This appears to be a favorable strategy for optimizing performance in this population (Franceschi et al., 2020).

### Loading Determination

A substantial portion of Brazilian Olympic sprint and jump coaches (47%) determined set loads based on movement velocity (e.g., using accelerometers or linear position transducers) (Thompson et al., 2020), while a similar proportion (26%) utilized one-repetition maximum (1RM) tests or varied the loading determination method according to athletes’ preferences or characteristics (i.e., “athlete dependent”). The third and fourth most frequently used methods were, respectively, “subjective guess” and “trial and error” (16% for both) and “predicted 1RM” and “rating of perceived exertion” (11% for both). Interestingly, the percentage of sprint and jump coaches utilizing “movement velocity” to define set loads (47%) was higher than that observed in studies performed with S&C coaches from professional cricket (~35%) and soccer (40%; coaches from 18 countries) (Weldon et al., 2021a; Weldon et al., 2021b) and represented almost double value of that reported for Brazilian soccer S&C coaches (24%) (Loturco et al., 2022). High precision of the “velocity-based method” aligned with the possibility of controlling training sessions in real-time and monitoring the variations in strength-power levels that can occur daily have contributed to the increased popularity of this approach among sprint and jump coaches (Pareja-Blanco and Loturco, 2022). Indeed, these technical and practical aspects are extremely relevant in explosive sport disciplines, where strength and power production are of paramount importance (such as sprint and jump events) (Loturco et al., 2019).

Another point to note is that elite Brazilian soccer S&C coaches reported using 1RM tests less frequently (8%) than Olympic sprint and jump coaches in this study (26%) (Loturco et al., 2022). This difference may be easily justifiable by three basic factors: 1) the long time it takes to complete a 1RM test with multiple athletes (as in soccer) (Loturco et al., 2022), 2) the traditional use of 1RM measurements and training sessions with heavy and very-heavy loads in track and field disciplines, and hence 3) the ability of top-level sprinters and jumpers to properly perform maximum strength tests (Haugen et al., 2019b). Similarly, the solid and extensive resistance training background of this highly specialized population allows coaches and athletes to select and define appropriate methods of load determination throughout the entire training season, which also explains the high portion of “athlete dependent” responses obtained (26%). All the remaining responses (i.e., “subjective guess”, “trial and error”, “predicted 1RM”, and “rating of perceived exertion) achieved frequency rates equal or inferior to 16% of the total of responses reported herein and are possibly related to personal coaching preferences or lack of appropriate technology (e.g., devices to measure, for example, barbell velocity).

### Resistance Training Programming

Most sprint and jump coaches in this study (52.6%) reported implementation of more flexible training programs, adjusting training content, loads, and strategies according to training responses or objective needs (e.g., qualification for participation in international track and field competitions). Somewhat curiously, only 31.6% of them preferred to utilize a fixed and periodized training routine, previously defining some competitions as “most important events”, where sprinters and jumpers had to achieve their peak performance. Some coaches offered additional comments to this question mostly indicating that the *“high number of sequential competitions over a considerable short-period of time in the second trimester of the year”* combined with *“the necessity of meeting the high qualification standards for international competitions (e.g., Diamond League Meetings and World Championships)”* required coaches *“to frequently change and adjust their training practices, according to the needs and objectives of athletes”*. In this regard, for example, if an elite sprinter achieves the qualification time for these key competitions at the beginning of this congested competition cycle, coaches may program the subsequent training phases with a focus on these international events. In contrast, when these main goals are not met at earlier stages, coaches are required to rethink and readjust their training strategies, taking into consideration the competitive performance and specific needs of their athletes.

Other additional comments were related to the current evidence contesting training periodization (Afonso et al., 2017; Afonso et al., 2019; Kataoka et al., 2021; Kiely, 2012; Kiely, 2018) that, among other things, *“may encourage coaches to search for more modern, effective, and feasible training approaches”*, which *“could be more adapted to the current competitive scenario”*. Although these statements were based on personal opinions and subjective perceptions, they certainly reflect a trend towards an important change in some traditional training paradigms, primarily driven by the necessity of creating more realistic (and “agile”) training schemes (Conceiçao and Affonso, 2022; Jovanović, 2020; Kiely, 2012; Loturco and Nakamura, 2016). A similar tendency was observed in a survey conducted with S&C coaches who worked in Brazilian elite soccer – a team-sport discipline that is even more affected by the congested fixture schedules, successive matches in different tournaments, and frequent journeys across the country (Loturco et al., 2022). Nevertheless, it is worth noting that the traditional periodization model is still widely and extensively used by coaches from various sports (Weldon et al., 2020). Hence, alternative training methods and concepts should not only be effective and viable in contemporary sport contexts, but also ensure their acceptance by the coaching community.

### Interval Between Resistance Training Sessions, Sport-Specific Training Sessions, and Official Competitions

The majority of Brazilian Olympic sprint and jump coaches programmed resistance training sessions on the same day or 24 h after specific training sessions (32% of coaches, in both cases). A smaller proportion of coaches implemented recovery intervals equal or superior to 48 h (16%, for both responses) or equal to 36 h (5%). Regarding the time between resistance training sessions and competitions, a significant number of coaches (42%) adopted intervals greater than 48 h, with a minority using intervals of 24 h (21%), 36 or 48 h (16%), or training “on the same day as competition” (5%). Lastly, most sprint and jump coaches (68%) provided more than 48 h of recovery intervals between sport-specific training (i.e., track and field training) and competition, followed by 48 h (16%), 24 and 36 h (5% for both), and training “on the same day as competition” (5%). In summary, the most common intervals applied between resistance training sessions, sport-specific training, and competitions were: ≤24 h between resistance and sport-specific training sessions; and >48 h between resistance training sessions and competitions and/or sport-specific training and competitions.

The findings obtained in this survey are difficult to compare with studies performed with other sports (e.g., soccer and cricket) as for sprint coaches, the sport-specific training session is, essentially, a sprint-specific training session. Nonetheless, it seems that recovery intervals ≤24 h between strength and power training and sport-specific training are commonplace between S&C coaches of several sports (Loturco et al., 2022; Weldon et al., 2021a; Weldon et al., 2021b). This may be another indication of the influence of congested fixture schedules on modern sports. Other surveys on sprint coaches have not reported or enquired about specific recovery intervals between distinct training sessions (Bolger et al., 2016; Healy et al., 2021; Jones et al., 2009). However, all of them highlight, in different forms and to different extent, the physically demanding characteristics of sprinting and the key role played by strength-power capacities in (and their potential transference to) elite sprint performance. As a consequence, apparently, Brazilian Olympic sprint and jump coaches prioritize longer recovery intervals between resistance or sport-specific training sessions and competitions to optimize the recovery processes and functional readiness, and thus, the subsequent performance of their athletes (Howatson et al., 2016; Thomas et al., 2018).

### Resistance Training Modalities and Exercises

Variable resistance training was extensively employed by 79% of Brazilian Olympic sprint and jump coaches. They also used, to similar extent, eccentric (63%), concentric (58%), and machine (53%) resistance training modalities. Isometric and isoinertial exercises were implemented by 37% and 16% of coaches, respectively. The high usage of variable resistance training among S&C coaches was also observed in professional team-sports from different leagues and countries (i.e., ~60% in soccer and 76% in cricket) (Loturco et al., 2022; Weldon et al., 2021a; Weldon et al., 2021b). However, for these coaches, concentric and eccentric training modalities were still the most common forms of resistance training. Two factors may explain these results: 1) the scarcity of appropriate track and field training facilities throughout the country (i.e., Brazil), which often means that coaches need to develop and use alternative training approaches (e.g., elastic bands as resistance); 2) the proven effectiveness and practical aspects of variable resistance training, especially in terms of maximum strength development, either in isolation or in combination with more traditional training strategies (e.g., free-weight exercises) (Loturco et al., 2020; Soria-Gila et al., 2015).

In contrast, the use of eccentric and concentric training modalities is in line with that reported for Brazilian soccer S&C coaches (~60– 65%), which is easily understood by examining the traditional application and wide popularity of these methods (Loturco et al., 2022; Weldon et al., 2021a). Interestingly, sprint and jump coaches declared that they more regularly utilized machine-based resistance exercises (i.e., 53% versus ≤37% in other sports) (Loturco et al., 2022; Weldon et al., 2021a, 2021b), which is probably related to their rationale for “choosing the most important exercises for their athletes” (Healy et al., 2021). In this regard, Healy et al. (2021) used a thematic analysis approach to determine why sprint coaches prioritized some exercises, identifying five distinct reasons: performance adaptations, practicality, muscle groups, movement characteristics, and similarity to sprinting. Importantly, the most prominent theme that emerged for selecting traditional exercises was “muscles/muscle groups”. In practical terms, sprint coaches primarily considered the targeted muscle groups when prescribing strength exercises. Taking into account the complex nature of sprinting (Cronin and Hansen, 2005; Loturco et al., 2017; Loturco et al., 2019; Markovic et al., 2007) and the large number of muscles involved in these short maximal efforts, it is plausible that these coaches are more likely to include certain machine-based exercises (e.g., multi-hip, leg-extension, and leg-curl machines) in their training routines, to specifically increase, for example, hip and knee extension and flexion torque (Blazevich and Jenkins, 1998; Miller et al., 2021; Taylor et al., 1991). The lower use of isometric and isoinertial training modalities (compared to the abovementioned methods and/or other sports) is clearly related to the dynamic characteristics of sprint and jump events (which reduce the importance and specificity of isometric actions) and the absence of isoinertial training devices in Brazilian training facilities.

Squats along with different variations of this exercise, Olympic weightlifting and its derivatives, as well as ballistic exercises were reported to be the most commonly used, appearing at least once as “main exercises” and at least three times among the three most frequently used exercises in the five sprint and jump coaching ranks. The hip thrust exercise was mentioned four times among the five most important exercises within the five ranks, while the other exercises (e.g., lunges, calf raises, leg extension, leg curls, core exercises, stiff-leg deadlifts, and the bench-press) appeared in a very varied and inconsistent manner. The frequent use of squats and Olympic weightlifting exercises is a common practice among professional coaches from different sports (Weldon et al., 2022) and, specifically in some track and field disciplines, this regular utilization seems to be highly encouraged (Bolger et al., 2016; Cissik, 2010). Higher levels of lower limb force production, power, and explosiveness have been considered key aspects for successful sprint performances which, per se, support the prescription of squat-based movements and Olympic lifts for elite sprinters and jumpers (Bolger et al., 2016; Cissik, 2010; Haugen et al., 2019b). Likewise, the unique characteristics of ballistic exercises, i.e., movements that allow for continued acceleration throughout the entire range of motion (Cormie et al., 2011b; James et al., 2018), certainly influence coaches’ selection criteria, as maximum acceleration capacity and the ability to apply substantial amounts of force at higher velocities (i.e., muscle power) also play determinant roles in explosive track and field disciplines (Aoki et al., 2015; Haugen et al., 2019a; Loturco et al., 2018a). Lastly, the common use of the hip-thrust exercise can be related to the easy and practical execution of this barbell-based movement and its close relationship with and potential effects on acceleration and top-speed performance (Dello Iacono and Seitz, 2018; Loturco et al., 2018a; Ribeiro et al., 2020). The less frequent prescription of the other exercises may suggest that they are primarily employed to suit the individual needs of athletes (i.e., tailored training plans) or personal coaching preferences.

### Speed Development and Plyometrics

As expected, a wide variety of training strategies was regularly used by Brazilian Olympic sprint and jump coaches for speed development. Maximum speed sprinting was the most common speed training method, being prescribed by 84% of sprint and jump coaches. The reasons behind the consistent adherence to this type of training are clear: for sprinters and jumpers, maximum sprint efforts represent the expression of very specific skills which are directly related to their competitive performance (Bridgett and Linthorne, 2006; Granić and Pavlović, 2021; Ngetich and Rintaugu, 2013). When implementing this training approach, coaches not only enhance the physical attributes of their sprinters and jumpers, but also improve and control their technical preparedness. Form running (i.e., technical sprinting drills), resisted running, overspeed running, and uphill/downhill running (utilized by 74%, 68%, 58%, and 53% of coaches, respectively) can also mimic, from a partial or a more general perspective, the typical motor pattern observed in traditional sprints (i.e., unloaded flat sprints), which encourages their regular use as speed-specific training strategies (Haugen et al., 2019b; Pareja-Blanco et al., 2020; Whelan et al., 2016). Strength training, sport-specific movements (i.e., isolated sprint movements with added resistance), complex training, and interval training methods seem to work as complementary training activities for optimal speed development, being used for this purpose by 42%, 37%, 21%, and 16% of coaches, respectively.

In line with previous studies conducted with similar or different coaching populations (Loturco et al., 2022; Weldon et al., 2022), a great portion of coaches surveyed here (74%) declared using plyometrics to increase sprint speed, with 89% of them considering speed development as the main reason for prescribing plyometric training sessions for top-level sprinters and jumpers. The second and third most important reasons, almost in similar proportions, were improving jumping ability and lower-body power (68% and 63%, respectively), whereas injury prevention, total-body, and upper-body power, were indicated by 26%, 16%, and 11% of coaches, respectively. Indeed, the effectiveness of plyometric training in enhancing speed performance is well supported in the literature, with several variations in training implementation, specific effects, and exercise types (Makaruk et al., 2020; Ramirez-Campillo et al., 2020). For example, while horizontally-directed jumps (e.g., broad jumps and alternate leg bounding) can be more recommended to improve the initial phases of sprint running (i.e., acceleration phase), vertically-directed jumps with relatively shorter ground contact times (e.g., in-place pogo jumps) seem to be more applied for developing top-speed qualities (Clark, 2018; Loturco et al., 2015c; Makaruk et al., 2020). Improving jump and lower-body power performance (which are, in fact, interrelated capabilities) may also be considered expected responses in a sample composed of elite sprint and jump coaches (Loturco et al., 2015a). Although less frequent, the other reasons for prescribing plyometric exercises (i.e., injury prevention in multi-intervention training programs, total-body and upper-body power development) are supported by a solid body of research and are commonly reported in surveys conducted with other sports (Hübscher et al., 2010; Loturco et al., 2022; Mcguigan, 2017; Weldon et al., 2021a). The majority of coaches implemented plyometric training in the preparatory or the competitive period (68% and 63%, respectively), although 37% of them prescribed plyometrics throughout the entire training season. These discrepancies are likely to be due to different training methodologies, since, as mentioned above, 52.6% of coaches in this study reported implementing more flexible training programs (instead of adopting a fixed and periodized training scheme). Therefore, many coaches utilize a variety of plyometric drills since the earlier periods of the training cycle, combining these exercises with, for example, power-oriented (i.e., light or moderate loads moved at faster velocities) or maximum-strength-oriented exercises (i.e., heavy loads moved at slower velocities) during different training phases (Cormie et al., 2011a; Cormie et al., 2011b; Freitas et al., 2017; Mcguigan, 2017).

In general, Brazilian Olympic sprint and jump coaches prescribed plyometric exercises on separate days and after resistance training sessions (53% of coaches, for both options) or used these exercises as part of complex training methods (42%). Only a minority (26%) prescribed plyometric training before resistance training, an aspect which was also noted in Brazilian soccer S&C coaches (Loturco et al., 2022). This is somewhat unexpected given the evidence that plyometrics is more efficient when executed under well-rested conditions (Chu, 1986; Comfort and Matthews, 2010), which could imply a higher number of coaches implementing this training strategy prior to (and not after) resistance training sessions. Nevertheless, it should be acknowledged that Olympic sprinters and jumpers train and perform at an exceptional level and, hence, coaches may utilize this sequential pattern (i.e., weight training + plyometrics) in an attempt to optimize training responses via post-activation performance enhancement (or other delayed potentiation responses) (Boullosa, 2021; Harrison et al., 2019).

Different types of jumps were commonly used by coaches, with emphasis on hurdle jumps, bounding, box drills, depth jumps, and multiple hops (used by 89%, 79%, 74%, 74%, and 68% of coaches, respectively). Assisted jumps (42%), standing jumps (21%), and upper-body plyometrics (21%) completed the diverse sets of plyometric exercises regularly applied by Brazilian Olympic sprint and jump coaches. The high utilization of hurdle and box jumps (including depth jumps) and multiple hops (e.g., ankle and single-leg hops) is commonplace among sprint coaches who believe that these exercises could, among other things, *“strengthen the legs and help with ground contact”, “improve reactive and eccentric strength”*, and *“apply resistance to sprinters on the track”* (Bolger et al., 2016; Healy et al., 2021; Whelan et al., 2016). *“Similarity to sprinting”, “applicability”* (i.e., low-cost and ease of implementation) and *“injury prevention/reduction”* are other reasons often mentioned by track and field coaches to justify the regular implementation of plyometrics (Healy et al., 2021; Husbands, 2013; Whelan et al., 2016). Thus, from a general perspective, the rationale of Brazilian coaches for choosing and prescribing these commonly used exercises (as well as assisted jumps and upper-body plyometrics) is probably based on traditional coaching views and perceptions, which is also supported by the existing evidence on the positive effects of plyometric training on sprint and jump performance (Bolger et al., 2016; Chu and Meyer, 2013; Husbands, 2013; Rimmer and Sleivert, 2000; Saez De Villarreal et al., 2012).

### Flexibility Development

Sprinters and jumpers were required to perform flexibility training after and before training by 37% and 32% of the coaches, respectively. Only a small portion of Brazilian Olympic coaches (11%) encouraged their athletes to execute these exercises during the specific training activities. Most of the coaches used dynamic stretching (84%) and more than half of them (53%) also prescribed active stretching and proprioceptive neuromuscular facilitation. Ballistic or more traditional static, passive, and isometric flexibility exercises were applied by 47%, 42%, 21%, and 11% of coaches, respectively. For 37%, 21%, and 5% of coaches, flexibility sessions lasted 11–15, 16–20, or 6–10 min, respectively, while for 26%, these sessions last >21 min. Utilization of dynamic stretching exercises by Brazilian Olympic sprint and jump coaches was higher than typically observed for other coaching populations (84% versus 75%, on average), which contrasts with their lower preference for static stretching (42% for our sample versus 90%, on average, for coaches from eight distinct sports) (Weldon et al., 2022). These marked differences in flexibility training strategies are partially predictable and possibly driven by the solid evidence to support these practices. Several articles have appeared in the last decade suggesting that coaches should be cautious when prescribing static stretching to athletes who compete in speed- and power-related sports, particularly when any (minimal) increase or decrease in performance may be relevant (which is a clear case for sprint and jump competitions) (Behm and Chaouachi, 2011; Perrier et al., 2011; Yamaguchi and Ishii, 2005). On the other hand, dynamic stretching may provide a potential stimulus to the neuromuscular system, thus improving (or, at least, not compromising) performance in activities that involve sprinting or jumping (Behm and Chaouachi, 2011; Perrier et al., 2011; Yamaguchi and Ishii, 2005). Although we recognize that this evidence is more applicable to warm-up activities (Behm and Chaouachi, 2011; Perrier et al., 2011), this certainly affects the decision-making process of sprint and jump coaches, guiding their practices especially before and during specific training sessions (i.e., track and field training). Finally, the average duration of flexibility training sessions does not differ substantially from that reported for other sports (i.e., ≤20 min) (Weldon et al., 2022), although it is possible to see a trend towards longer flexibility training sessions in our data (as only 5% of coaches prescribed 6–10-min sessions, versus 40% in other sports) (Weldon et al., 2022). Hence, flexibility training sessions for sprinters and jumpers comprised mostly dynamic stretching exercises, lasted between 6 and 20 min, and were performed before, after or during regular track and field training.

### Physical Testing and Well-Being Assessment

A substantial portion of Brazilian Olympic sprint and jump coaches (68%) regularly assessed their athletes all year round, whereas 21% of them preferred to perform physical tests only during the preparatory period. Few coaches (5%) opted to apply these measurements solely over preparatory or off-season periods. The regular use of physical tests throughout the year is expected in individual sport disciplines in which extremely high levels of physical and technical performance are necessary, as in sprint and jump events (Loturco et al., 2021a). Approximately, 70% of coaches surveyed here applied physical tests within different phases of the year, a percentage which is similar to that found in professional cricket coaches, but greater than that found, for example, in elite soccer S&C coaches from various countries (i.e., 46%) (Weldon et al., 2021b). However, there are important (but logical and specific) differences among the physical tests most frequently used in these sports. While professional cricket S&C coaches predominantly utilize cardiovascular endurance and body-composition measurements, sprint and jump coaches are more concerned with sprinting speed and muscular power tests (used by 74% and 63% of our sample). In contrast, only 11% of coaches surveyed in this study incorporated cardiovascular endurance assessments into their testing batteries (compared to almost 100% of cricket S&C coaches) (Weldon et al., 2021a). These discrepancies can also be observed when comparing our data with the practices employed by coaches from other sports who predominantly use body composition and muscular strength tests during their testing routines (86% and 75% of them, average data, respectively, compared to 58% in our coaching sample, for both measurements). Overall, sprint and jump coaches are primarily focused on monitoring and maintaining the speed and power levels of their athletes over the entire training season, which is directly related to the competitive performance of sprinters and jumpers (Haugen et al., 2019a; Haugen et al., 2019b; Loturco et al., 2015a). Anaerobic capacity and anthropometric assessments, along with acceleration tests were commonly used by 42%, 37%, and 32% of Brazilian Olympic sprint and jump coaches, respectively, and only 5% of them evaluated athletes’ flexibility. This can partly be attributed to the fact that flexibility exercises are mainly used during warming up or recovery routines, acting as complementary training activities, since flexibility is not a crucial determinant of sprint and jump performance (Haugen et al., 2019b; Perrier et al., 2011).

The vast majority of coaches (95%) frequently monitored the well-being of their sprinters and jumpers, which appears to be a common practice in elite sports (Weldon et al., 2021a; Weldon et al., 2021b). Nonetheless, there is a key difference here: track and field events are individual sport disciplines. As such, these coaches have continuous and very close contact with their athletes, allowing them to predominantly apply verbal methods to assess well-being through individual conversations. This explains why 79% of Brazilian Olympic coaches regularly utilize verbal questionnaires to evaluate sprinters and jumpers (versus, for example, 24% and 31% of professional cricket and soccer S&C coaches, respectively) (Loturco et al., 2022; Weldon et al., 2021a; Weldon et al., 2021b). The other well-being measurement tools (written and online questionnaires, and mobile applications) were employed by ≤11% of the coaching sample. Overall, Brazilian Olympic sprint and jump coaches evaluated their athletes continuously, prioritizing speed and power tests, and using verbal questionnaires to subjectively assess well-being at an individual level.

### Use of Technology Resources

Technology-based equipment was used by 79% of Brazilian Olympic sprint and jump coaches, with video analysis software being the most popular resource (used by 47% of coaches). During some specific training phases (i.e., close to competitions), some coaches may even utilize video analysis tools daily to adjust and refine movement technique and posture (Shih, 2017). Speed gates and mobile phone apps (to assess jump height and barbell velocity, for example) ranked as the second most common options (42%, for both devices), followed by jump mats and bar-velocity trackers (used by 37% and 32% of coaches, respectively). The more frequent usage of these devices is compatible with their preferences to frequently assess and monitor sprinting speed and muscular power. Body composition analyzers, force plates, and wearable technologies were used by ≤26% of Brazilian Olympic sprint and jump coaches. This is plausible, given that these technologies are much more expensive (especially in the Brazilian context), and less user-friendly and accessible than “simpler devices” (i.e., force plates and jump mats).

## Conclusions

The sample surveyed in this study had a solid international experience and comprised coaches who had already won several medals in the most important track and field championships around the world. In summary, the typical resistance training program prescribed by these coaches during the off-season period consisted of 2–4 sessions per week, which lasted more than 60 min, and included 3–4 sets of 4–6 or 10–12 repetitions per exercise. Across the competitive period, resistance training was usually performed twice a week, with sessions lasting between 46 and 60 min, with 3–4 sets of 4–6 repetitions per exercise. Training loads were predominantly determined by movement velocity, although several coaches revealed to use 1RM or “athlete dependent” methods (26% for both methods). The majority of coaches (52.6%) declared that they adopted more flexible training programs, adjusting training content and loads according to the individual demands, needs, or objectives of their athletes. Strength-power training was performed within the same day or 24 h after the specific track and field training sessions, using variable, eccentric, concentric, and “machine” training modalities. Squats along with different variations of this exercise, Olympic weightlifting and its derivatives, as well as ballistic exercises were reported to be the most frequently used exercises, while maximum speed sprinting was highlighted as the most common speed training method. Plyometrics were primarily used to increase sprinting speed, with various types of jumps being regularly used by coaches, especially hurdle jumps, depth jumps, bounding, box drills, and multiple hops. Flexibility training sessions usually included 6–20 min of dynamic stretching exercises, executed before, after or during track and field training. Most of coaches surveyed here opted to test their athletes all year round, with a special focus on sprint speed and power-related capacities. Athletes’ well-being was typically assessed by verbal questionnaires, in a personal and tailored context. Approximately 80% of Brazilian Olympic coaches utilized technological-based equipment to evaluate their sprinters and jumpers, with video analysis software, speed gates, mobile phone apps, jump mats, and bar-velocity trackers being the most regularly used tools. The S&C practices used by leading track and field coaches surveyed in this study may help practitioners and sport scientists create more effective research projects and training programs (Haugen, 2021).
